# A Challenging Case of Lemierre’s Syndrome With Central Nervous System Involvement and a Comprehensive Review

**DOI:** 10.7759/cureus.10131

**Published:** 2020-08-30

**Authors:** Suhas P Dasari, Harpreet Gill, Haley Bodette, Elizabeth Brandes, Pinky Jha

**Affiliations:** 1 Internal Medicine, Medical College of Wisconsin, Wauwatosa, USA

**Keywords:** lemierre's syndrome, fusobacterium, necrobacillosis, meningitis, abducens nerve palsy, sinusitis, cavernous sinus thrombosis, postanginal septicemia, septic embolism, f. necrophorum

## Abstract

Lemierre’s syndrome (LS) is a complex medical condition that is characterized by an acute oropharyngeal infection leading to thrombophlebitis of the internal jugular vein and an eventual metastatic spread to distant vital organs. This metastatic spread is from septic emboli and is most frequently seen in the lungs, kidneys, and large joints. Central nervous system (CNS) involvement in LS is extremely rare, and only a few cases of CNS involvement have been reported in the literature. For all cases of LS, early diagnosis and treatment are crucial, yet due to the rarity of CNS complications in LS, diagnostic patterns and treatment algorithms are not fully understood for this subset of presentations. In this report, we present a case of 19-year-old immune-competent female who presented with a Fusobacterium oropharyngeal infection that was complicated by suppurative sinusitis, cavernous sinus thrombosis, meningitis, and an abducens nerve palsy. Our patient was treated with broad-spectrum antibiotics, anticoagulation, and multiple surgical interventions. This case highlights the importance of an early diagnosis and a multidisciplinary approach in managing LS to minimize the mortality and long-term morbidity of this relatively rare and complicated multisystem disease.

## Introduction

Lemierre’s syndrome (LS), or human necrobacillosis, is a rare and life-threatening condition that is most frequently caused by infection by the anaerobe *Fusobacterium necrophorum*. It typically presents with a pattern of an initial oropharyngeal infection that leads to an internal jugular vein (IJV) thrombus formation and subsequent metastatic spread through septic emboli. The most frequent causal pathogen, *F. necrophorum*, is an obligate anaerobic gram-negative bacillus that is a commensal organism of the oropharynx and is seen in higher prevalence with increased pathogenicity in the flora of individuals who have had recurrent sore throats. It is an obligate anaerobic gram-negative bacillus. The earliest reported case of human necrobacillosis was in 1900 [1]. Andre Lemierre, whom the disease is named after, subsequently presented a cohort of 20 cases with postanginal septicemia in 1936, which proved to be fatal for nearly all of the patients [1]. His great contribution to the scientific community was the clarity with which he described the clinical constellation of symptoms that occur in LS [1]. Much later in 1980, the classic triad of LS was described by Vogel and Boyer. This was an oropharyngeal infection causing *F. necrophorum* bacteremia and eventually IJV thrombosis [2]. It is important to note that both a positive blood culture and imaging of IJV thrombosis are not required for the diagnosis of LS so long as one of the two is present. Atypical presentations also exist involving other pathogens and anaerobic bacteria that make the diagnosis and management more challenging.

The etiology of LS can vary, and the method of metastasis is debated. The infection usually begins in the oropharynx and typically undergoes hematogenous spread to the IJV through the lateral pharyngeal space. After becoming systemic, LS displays its common clinical characteristics such as sepsis, neck swelling, and a sore throat. Additionally, septic embolism is a defining feature of LS and is seen in the lungs, kidneys, and large joints; however, the central nervous system (CNS) is relatively well protected due to its upstream location relative to the IJV. As a result, few cases of CNS involvement have been reported. In general, CNS complications secondary to LS represent only 1-3.6 % of all cases [1,3].

Here we present a case of LS complicated by sinusitis, bacterial meningitis, cavernous sinus thrombosis, superior ophthalmic vein thrombosis, and a bilateral abducens nerve palsy following a *Fusobacterium* oropharyngeal infection. These rare and potentially fatal developments of LS require a multidisciplinary approach for prompt diagnosis and management for good outcomes. In our attempt to emphasize early recognition and treatment, we report this case of LS.

## Case presentation

**Figure 1:**
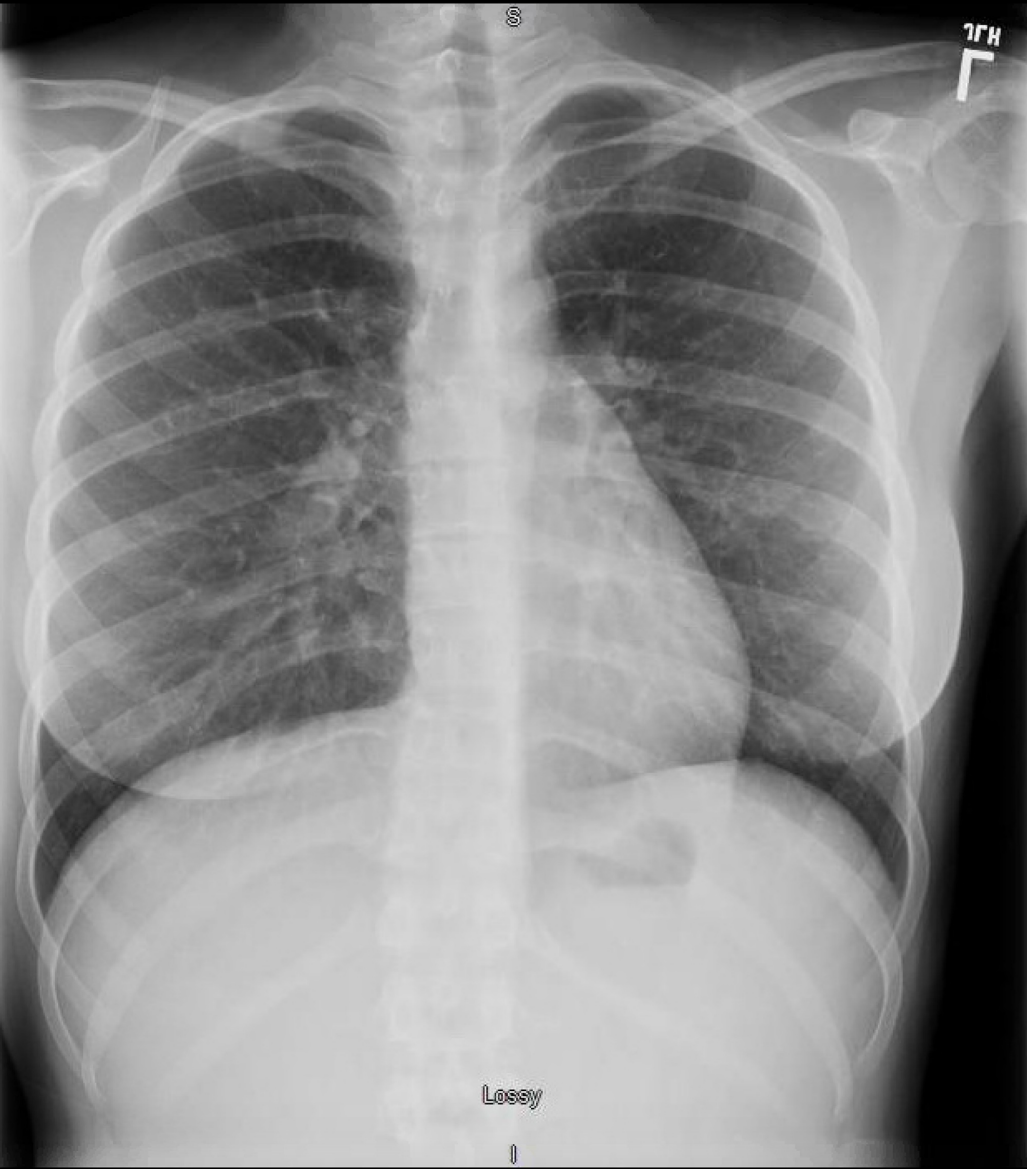
Chest X-ray (anteroposterior view) showing normal findings.

**Figure 2:**
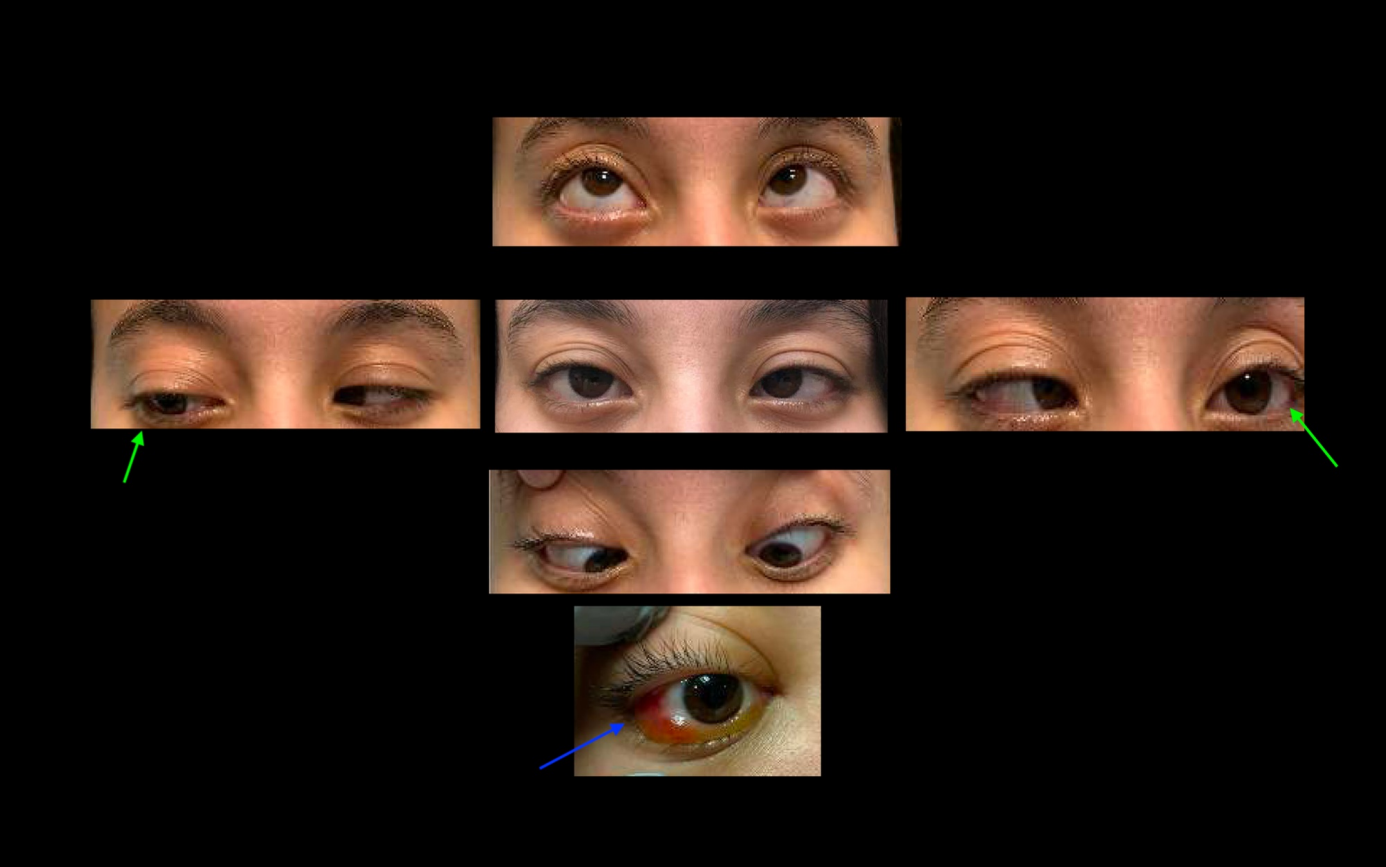
Eye examination showed right eye proptosis, chemosis (blue arrow), pain with extraocular eye movements, and bilateral abducens nerve palsy (green arrows, left-ward gaze worse than right-ward gaze).

**Figure 3:**
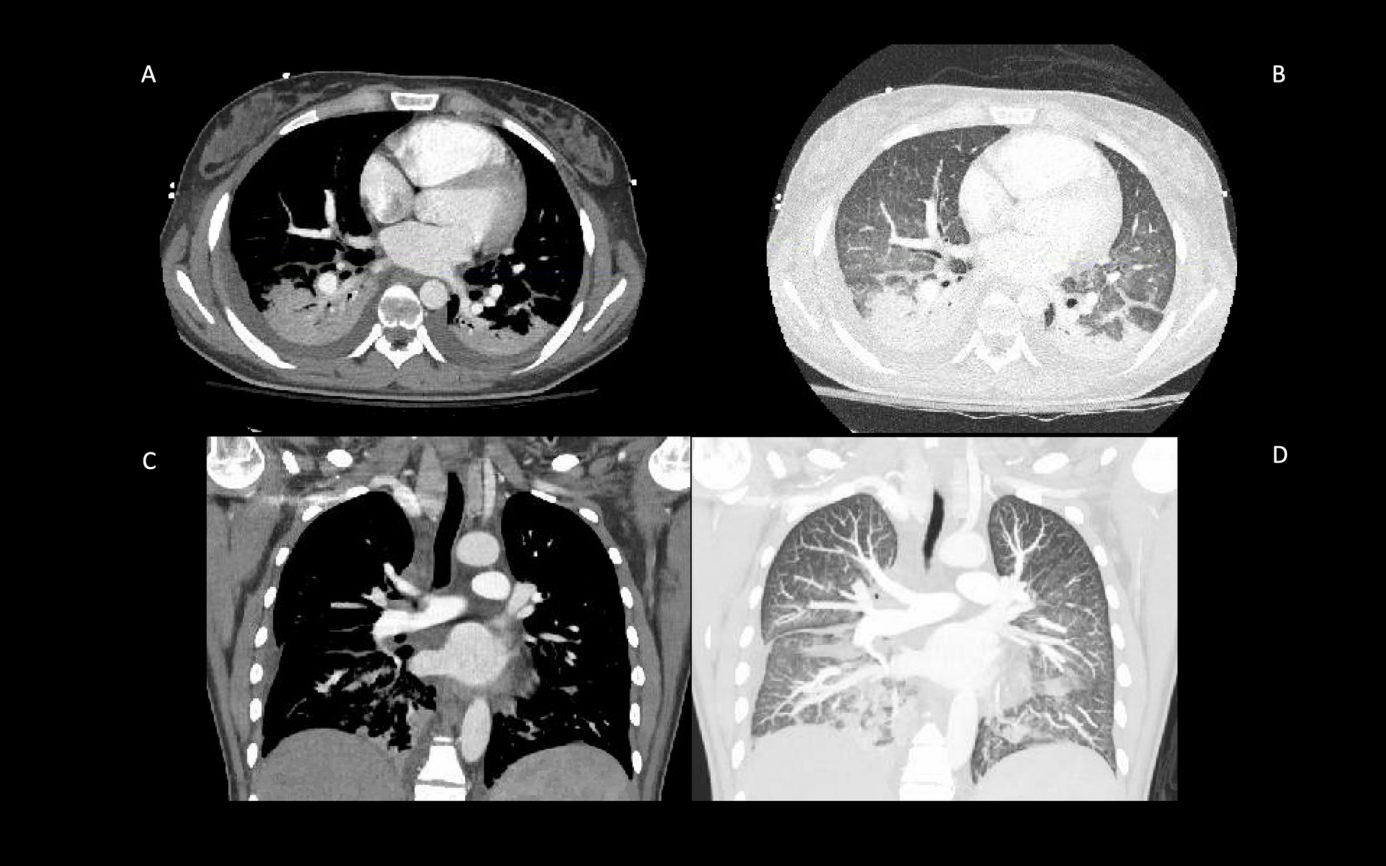
Axial CT without contrast (A), axial CT with contrast (B), coronal CT without contrast (C), and coronal CT with contrast (D) showing no abnormal findings.

**Figure 4:**
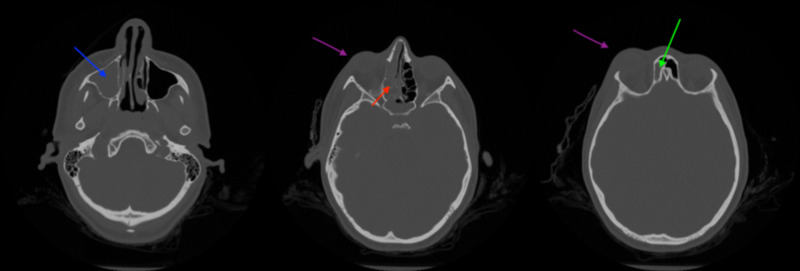
CT without contrast (axial sections) showing right proptosis (purple arrow) as well as complete opacification of the right maxillary sinus (blue arrow), right ethmoid sinus (red arrow), and right frontal sinus (green arrow).

**Figure 5:**
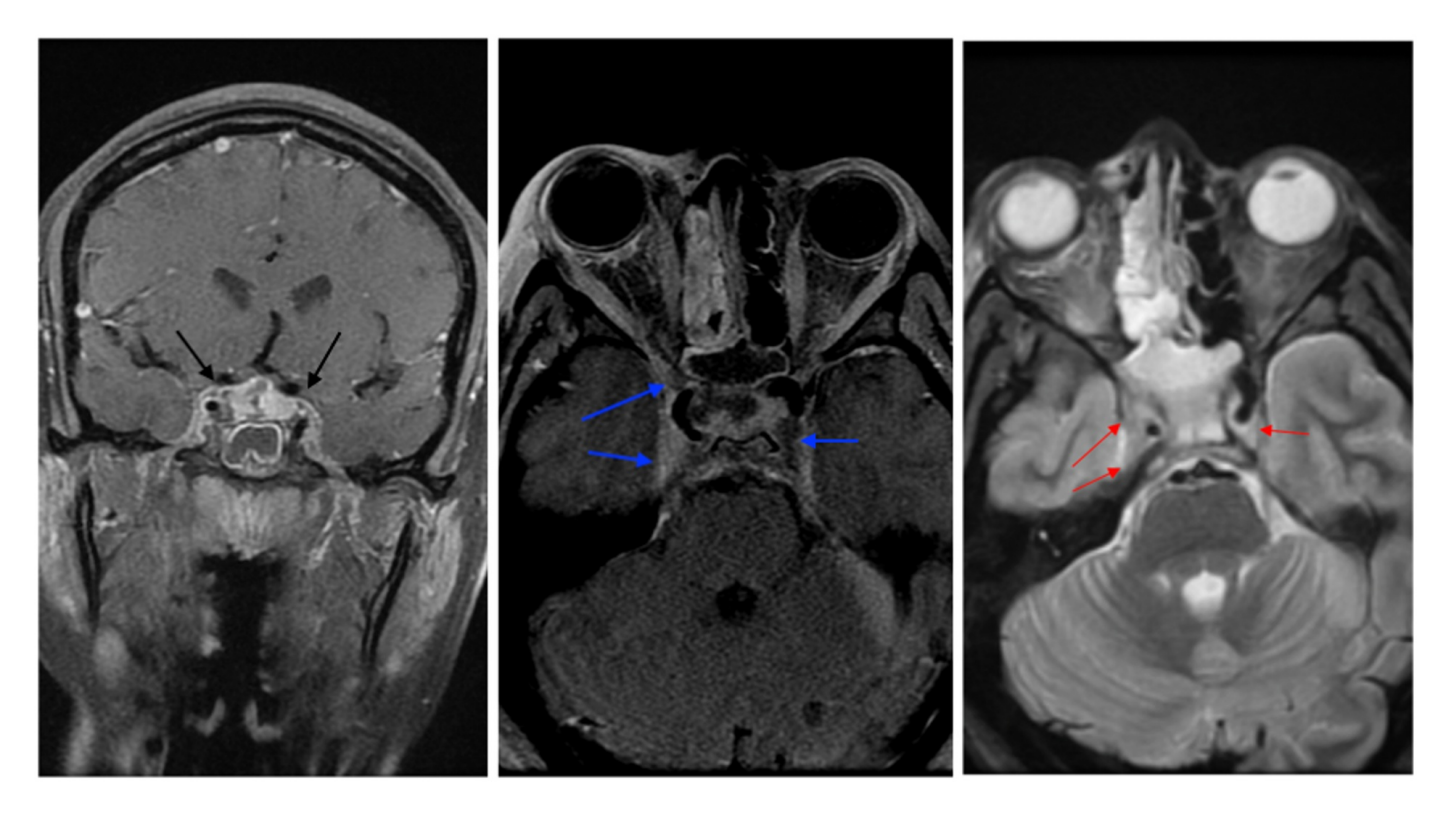
MRI showing filling defects indicating bilateral cavernous sinus thrombosis with the right side being greater than the left side (T1 contrast coronal: right, black arrows; T1 contrast axial: middle, blue arrows; T2 no contrast axial: left, red arrows).

**Figure 6:**
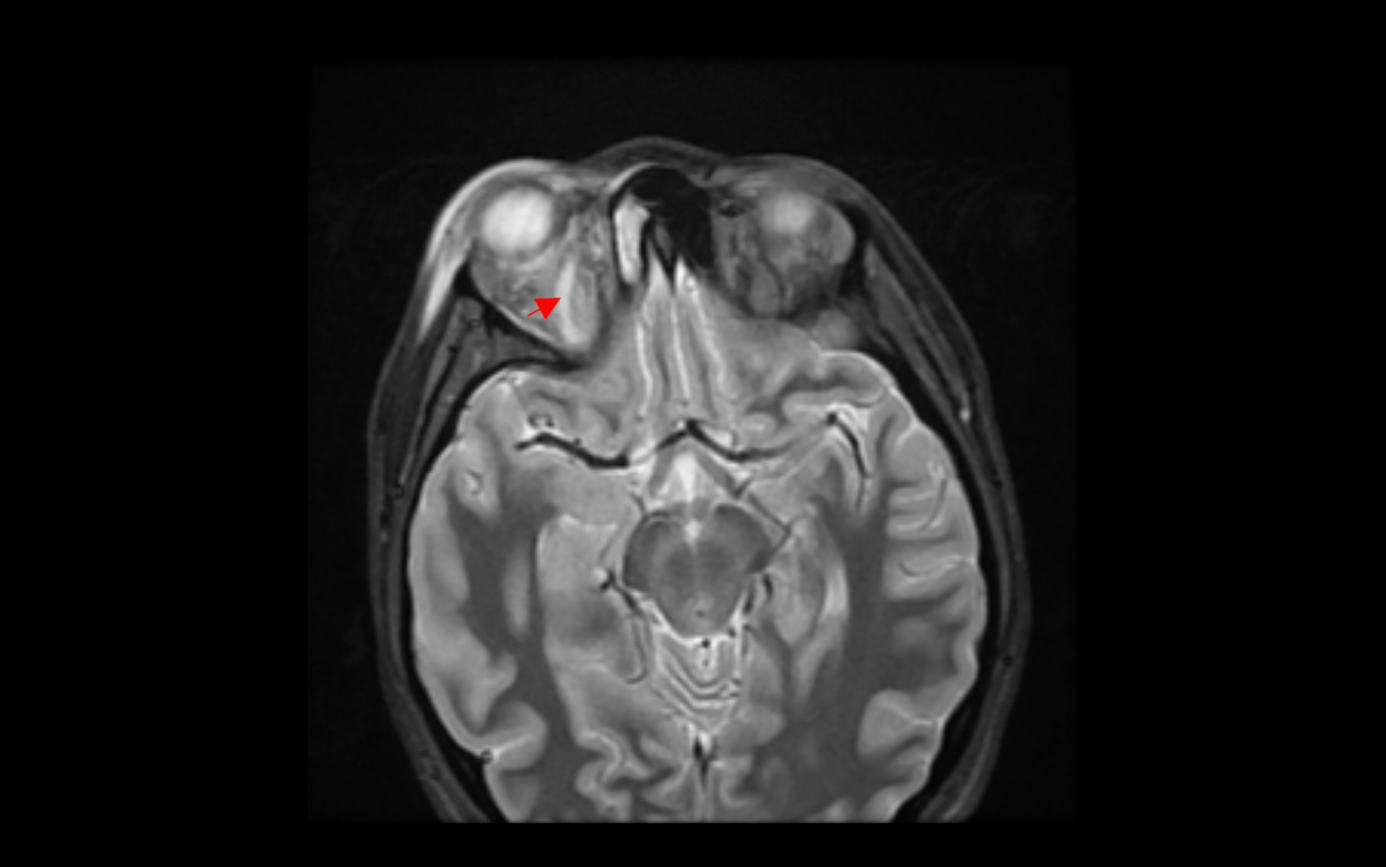
Axial STIR MRI without contrast showing thrombosis of the right superior ophthalmic vein with right orbital proptosis and enlargement of right lateral rectus. STIR, short tau inversion recovery

A 19-year-old female with a past medical history of asthma presented to urgent care with complaints of a severe sore throat, neck swelling, and fatigue for two days. The initial work-up showed a positive heterophile/EBV reflex test, a negative group A strep test, and a negative influenza A and B test. She was discharged with supportive care for infectious mononucleosis. Over the next week, her symptoms continued to worsen. She was taken to the emergency department for an episode of hemoptysis, fever, eye pain, and copious purulent drainage from her nose. Upon presentation, she was in distress due to a severe headache, photophobia, and blurred vision. She also endorsed having diplopia, bloody purulent nasal discharge, neck swelling, nausea, vomiting, and anorexia. She denied any recent travel, trauma, or history of neurosurgery. The patient was hypotensive (96/60 mm Hg), febrile (103 °F), and tachycardic (120 beats/minute) upon presentation. Her examination revealed sinus tenderness, nuchal rigidity, and right-sided cervical lymphadenopathy. The eye examination showed right eye proptosis, chemosis, pain with extraocular eye movements (particularly with upward gaze), and a bilateral abducens nerve palsy (Figure 1). The rest of her exam was unremarkable. Her labs upon admission showed a leukocytosis of 18 K/µL (4.0-10.5 K/µL), thrombocytopenia of 36 K/µL (140-450 K/µL), transaminitis with an aspartate aminotransferase (AST) of 56 U/L (0-32 U/L) and alanine aminotransferase (ALT) of 49 U/L (0-33 U/L), alkaline phosphatase of 164 U/L (30-115 U/L), total bilirubin of 4.07 mg/dL (0.0-120 mg/dL), C-reactive protein (CRP) of 192.10 (<10 mg/L), international normalized ratio (INR) of 1.3, D-dimer of 4.41 ug/mL (0.0-0.50 ug/mL), and procalcitonin of 90.71 ng/mL (0.0-0.25 ng/mL). A CT scan of the head, a chest X-ray, and blood cultures were ordered. Due to suspicion for meningitis, a lumbar puncture was performed in the emergency department. The lumbar puncture showed cloudy yellow fluid, white blood cells 2,069 cells/µL (0-4 cells/µL), 66% neutrophils, glucose of 56 mg/dL (40-75 mg/dL), and protein of 190 mg/dL (15.0-45.0 mg/dL). This was suggestive of bacterial meningitis. Aerobic and anaerobic cultures of her cerebrospinal fluid (CSF) were negative. A CT of the head showed bilateral cavernous sinus thrombosis, right superior ophthalmic vein thrombosis, and opacification of the right frontal, maxillary, and sphenoid sinuses (Figure 2). The chest X-ray and chest CT were unremarkable (Figures 1, 3). The patient was admitted to the intensive care unit for the management of sepsis. An extensive initial work-up, including HIV, rapid plasma regain (RPR), and viral hepatitis serologies, was negative, but the preliminary blood cultures would eventually grow gram-negative rods. Based on the examination, abnormal laboratory findings, and abnormal imaging findings, numerous departments were consulted, including infectious disease, otolaryngology, neurosurgery, and ophthalmology. She was then started on broad-spectrum IV antimicrobials including linezolid, meropenem, acyclovir, and doxycycline along with heparin for anticoagulation. An MRI of the brain supported her CT scan findings of a cavernous sinus thrombosis, thrombosis of the right superior ophthalmic vein with concomitant right orbital proptosis, retro-orbital congestion, and enhancement with enlargement of the right lateral rectus (Figures 4-6). When her blood cultures came back positive for *F. necrophorum*, the antibiotic regimen was narrowed to ceftriaxone and metronidazole. Of note, the CT scan and ultrasound of the neck were negative for jugular venous thrombophlebitis. An ultrasound of the liver and spleen was performed to rule out septic emboli. It was negative for any abscesses but did show splenomegaly (14 cm). The patient required multiple right sinus surgeries including maxillary antrostomy, ethmoidectomy, sphenoidectomy, and septoplasty during her stay. The culture from the tissues obtained from her nasal sinuses after the procedure also grew *Fusobacterium*. The ophthalmology service performed daily vision checks and treated her right eye prophylactically with erythromycin due to lagophthalmos. For her cavernous sinus and superior ophthalmic vein thrombosis, she was continued on heparin that was later transitioned to warfarin. On day 6 of admission, the patient showed significant clinical improvement with decreased headache, reduced eye pain, improved visual acuity, decreased proptosis, and increased extraocular motility. However, she continued to have medial deviation of her left eye. She was discharged home on day 12 with IV ceftriaxone and oral metronidazole for a two-week course with three months of warfarin and follow-up appointments scheduled one week after discharge. After completing her IV antibiotic course, the patient was transitioned to amoxicillin/clavulanate for an additional two weeks. At her ENT follow-up, a nasal endoscopy was performed that showed well healing surgical sites. At her oculoplastic follow-up, she continued to show left eye medial deviation that was unchanged from when she was discharged. Furthermore, she continued to endorse blurry vision and diplopia that was improving but denied eye pain or pressure. At this point, she was referred to neuro-ophthalmology for further evaluation of her abducens nerve palsy.

## Discussion

Here we report a complex case of LS caused by *F. necrophorum* with rare and severe neurological complications including meningitis, superior ophthalmic vein thrombosis, and cavernous sinus thrombosis, resulting in a bilateral abducens nerve palsy.

LS is a medical emergency that is caused by anaerobic oropharyngeal bacterial infection (commonly *Fusobacterium*) leading to bacteremia, thrombophlebitis of the IJV, and septic embolism usually in young, previously healthy adults. While thrombophlebitis of the IJV is a classic feature of LS, it is not required to be present for the diagnosis of the condition. In our review of the literature, we found many case reports of LS with positive cultures for *F. necrophorum* despite having no imaging showing thrombosis in the veins of the head and neck. According to Olson et al., the modern diagnostic criteria include a history of anginal illness of the oropharynx within the preceding four weeks, evidence of metastatic lesions in the lungs or another remote site, and evidence of IJV thrombophlebitis or isolation of *F. necrophorum* from the blood or another sterile site [1,3,4]. Although our patient did not have IJV thrombophlebitis on imaging, she met the criteria for LS based on positive blood culture, preceding anginal illness of the oropharynx, and metastatic disease from septic emboli.

LS is a rare disease with an estimated global incidence of 1 case per 1 million people per year [1,5]. It is an underrecognized and underreported condition because of confusing characteristics, resulting in a low incidence. The burden of this disease tends to fall on the young, with approximately 90% of cases in individuals 10-35 years of age [6]. Mortality for the disease has improved significantly since the first reported cases due to the use of antibiotics. A 2016 five-year retrospective review of 137 cases reported a case fatality rate around 2%, showing further improvement from the 5% typically seen in reviews from 1980 to 2010 [1,3,5].

We conducted a review of previous studies starting with Lemierre’s original report in 1936 to determine the number of reported cases. This is outlined in Table 1. In their 1972 review, Bartlett and Finegold did not find any reported cases of LS between 1950 and 1960 [7]. During this time, LS became known as the “forgotten disease”. In the 1970s, cases of LS began reappearing in part due to increased diagnostic imaging. While the incidence for LS is low, there is some evidence that it is increasing [8]. While reviewing previous studies, we found that more recent studies tended to report a higher incidence than 1.0 per 1.0 million people. For example, a three-year prospective study of *F. necrophorum* infections in Denmark reported the incidence of LS at 3.6 cases per million inhabitants with a staggering 14.4 cases per million in people 15-24 years of age [8]. A more recent Swedish retrospective study also looking at *F. necrophorum* found the incidence of infection increased from 2.9 to 5.0 cases per million people, and 34% of individuals in this study developed LS [9]. Possible causes of this increase could be due to better reporting, different study design, location, and increased awareness of LS.

**Table 1:** Literature review of LS between 1936 and 2019 LS, Lemierre’s syndrome; CNS, central nervous system

Study (Year)	Dates Reviewed	Cases	Inclusion Criteria	CNS Findings
Lemierre (1936) [10]		20		
Sinave et al. (1989) [11]	1974-1988	38		
Armstrong et al. (2000) [12]	1990-1999	53	A recent pharyngeal infection; positive culture for F. necrophorum, thrombosis of internal jugular vein, or metastatic infections	
Riordan (2007) [1]	1970 – 2007	222	Hybrid of clinical and microbiological criteria in English and French databases including positive culture for F. necrophorum; internal jugular vein thrombosis; metastatic lesions	3.5% (n=9) cases with brain abscesses from F. necrophorum; 7.5% (n=19) cases of meningitis from F. necrophorum; 41% of otogenic F. necrophorum leads to meningitis; 3 LS cases with meningitis but from Fusobacterium ssp. or other anaerobes (1.3%)
Karkos et al. (2009) [5]	1950-2007	114	Positive culture for Fusobacteria; radiological evidence of thrombophlebitis	30% had complications in the brain (meningitis, epidural/subdural abscess, cavernous/sigmoid/transverse/lateral sinus thrombosis, and stroke); 5% had ophthalmic complications; 3% had lower (XI and XII) nerve palsy
Kuppalli et al. (2012) [13]	1974-1999	91	Internal jugular vein thrombosis; findings of F. necrophorum or other implicated bacteria	24% (n=22) had CNS complications; 13% (n=3) had cranial nerve palsy
Johannesen and Bodtger (2016) [3]	2010-2015	137	A recent pharyngeal illness; complicated by septic emboli; thrombosis of the internal jugular vein or findings of F. necrophorum in blood cultures	3.6% (n=5) had unspecified CNS complications.
Nygren and Holm (2019) [9]	2010-2017	104	A review of Swedish cases from all microbiology departments in the country; a recent oropharyngeal infection; development of thrombosis or signs of septic embolism; invasive infection due to F. necrophorum	1.9% (n=2) had unspecified CNS complications.

We also reviewed cases of LS that resulted in CNS complications. CNS complications secondary to LS are an extremely rare presentation because the IJV is downstream of the cerebral veins, protecting the CNS. In their literature review and presentation of a case with CNS complications, Kuppalli et al. found a total of 22 CNS-associated LS cases from 1980 to 2010 [13]. The most common symptom was meningitis, which was present in seven cases. Other symptoms included encephalitis, cerebral infarctions, brain abscesses, and septic emboli [13]. The severity of these symptoms resulted in an increased mortality rate of 18%, with an additional 30% of cases experiencing long-term complications. These outcomes highlight the importance of identifying LS and initiating treatment. In 2016, a five-year retrospective review by Johannesen and Bodtger found that CNS complications occurred in only 3.6% of the 137 cases identified [3].

LS starts as a seemingly harmless oropharyngeal infection but can rapidly progress to cause disseminated illness as the pathogenic organism is able to invade through protective mucosal barriers due to localized inflammation, trauma, or tissue damage. After entering the vasculature of the head and neck, the bacteria are able to create a thrombogenic environment and can spread through septic emboli to distant organs [14]. The lungs are the most common site for septic embolism, but metastatic infection can also manifest as septic arthritis, osteomyelitis, meningitis, pericarditis, and hepatic abscesses [15].

In our patient, metastatic spread was seen in the brain. While CNS involvement is rare, when present, it can cause meningitis, brain abscesses, cranial nerve palsies, and cavernous sinus thrombosis. Meningitis is a particularly rare complication of LS (1.3% of LS cases) [1]. Additionally, the meningitis associated with *F. necrophorum* is aggressive with a purulent CSF (WBC count median: 1,100/uL) and a poor prognosis that included more than 60% of cases resulting in death, permanent cranial nerve palsies, or hemiparesis [1]. Our patient presented with a purulent cloudy yellow CSF that had a WBC count of 2,069/uL, which nearly doubled the expected mean reported in the literature for LS complicated by meningitis. However, like the literature reported, patients with *F. necrophorum* meningitis tended to have a poorer prognosis including permanent cranial nerve palsies, and, unfortunately, our patient had not fully recovered from her bilateral abducens nerve palsy at the time of discharge.

While rare, cases of *Fusobacterium* meningitis have been reported in the literature. A literature review by Kuppalli et al. in 2012 showed seven reported cases of LS complicated by *Fusobacterium* meningitis [13]. Of these cases, five were males and two were females, with age ranging from 5 to 26 years. These patients suffered from a variety of other complications including venous sinus thrombosis, mastoiditis, cerebral and carotid artery stenosis, cranial nerve palsies, hemiparesis, dysarthria, Horner’s syndrome, and subdural empyema. Like many of the other cases that report CNS complications of LS, a majority of the cases in this review by Kuppalli et al. had poor outcomes. Of the seven patients, three were cured, one relapsed with LS after 14 months, and three had residual cranial nerve complications including vertical diplopia, sluggish right pupil, homonymous hemianopsia, and bilateral facial hypoesthesia [13]. In regard to our case, the patient was discharged with an abducens nerve palsy and diplopia. This follows the pattern set forth by the outcomes in the cases reviewed by Kuppalli et al. in 2012, where the patient suffering from meningitis was likely to have a poor outcome or unresolved neurological deficit. Although it is a rare complication of LS, meningitis is a marker of poor outcomes in LS, and these cases illustrate the importance of promptly recognizing and treating patients suspected of having LS and CNS complications.

In addition to *Fusobacterium* meningitis, our LS case was further complicated by a cavernous sinus thrombosis. A similar case of LS associated with an infectious cavernous sinus thrombosis and septic meningitis was reported by Shibuya et al. in 2012 [16]. Septic thrombosis of the cavernous sinuses is a potentially lethal condition that requires early recognition and treatment to minimize serious morbidity and mortality. The cavernous sinuses are located on either side of the sella turcica and are highly vascularized, leaving them susceptible to septic embolisms from localized infections of the head and neck [17]. Furthermore, the anatomical proximity of cavernous sinuses to cranial nerves III, IV, V, and VI is of great clinical importance [17]. Cavernous sinus thrombosis can present with multiple clinical features including fevers, proptosis, cranial nerve palsies, visual changes, headaches, and nuchal rigidity [18]. It is diagnosed by imaging (CT or MRI) and is frequently associated with a positive blood culture and CSF pleocytosis. Early treatment with IV antibiotics and anticoagulation can prevent fatal complications [18]. Surgical drainage of the cavernous sinus is almost never performed, but surgery may be essential for the management of the underlying sinusitis or oral infection/abscess [17].

Unfortunately, LS is difficult to diagnose due to its lack of pathognomonic symptoms and the rarity of the condition. This is particularly problematic as delays in treatment can double the mortality [3]. This leads to a delayed diagnosis until later in the disease course when IJV thrombosis is seen on imaging, metastatic spread becomes evident, or *F. necrophorum* is eventually identified on a blood culture. The first clinical symptom is usually nonspecific sore throat that can be mistaken for an infectious mononucleosis as was the case with our patient. Beyond clinical symptoms, labs are crucial for rapidly identifying LS. Inflammatory markers like CRP and ESR (erythrocyte sedimentation rate), imaging from ultrasound, CT, or MRI, and blood cultures prior to initiating antibiotics can be used to determine the diagnosis. To locate sites of septic embolism and evidence of IJV thrombosis, chest X-ray and ultrasound of the neck can be used for initial work-up, but contrast-enhanced CT scan and MRI are the most accurate tools for identifying the location of thrombus formation and septic emboli [1,3]. Blood cultures can be difficult and misleading in the early course of LS. This is because *F. necrophorum* has a long incubation time, leading to falsely reassuring sterile blood cultures. Additionally, there is molecular evidence, using PCR (polymerase chain reaction), to suggest that *F. necrophorum* has been the causal organism in culture-negative cases reported in the literature, suggesting that a negative culture does not necessarily exclude LS form the differential diagnosis [1,3,4].

Treatment of LS is three-pronged and is based on aggressive antibiotic treatment, anticoagulation therapy, and surgical intervention [19]. Delaying antibiotics may increase mortality and morbidity in patients with LS. A combination of ceftriaxone and metronidazole was used for treatment in this case as it provides coverage for both *F. necrophorum* and oral streptococci. Studies have shown that all strains of *F. necrophorum* are sensitive to metronidazole, ticarcillin-clavulanate, cefoxitin, amoxicillin/clavulanate, and imipenem, whereas resistance to erythromycin is relatively common in 15-22% of cases [1]. Metronidazole appears to be the best antimicrobial for treating human necrobacillosis as it has good oral bioavailability, excellent coverage of *Fusobacterium* species, and strong tissue and CNS penetration [1]. When metronidazole is combined with a carbapenem or piperacillin/tazobactam, it has a 98% success rate with an average treatment plan of four weeks [3]. If patients improve on two to three weeks of IV antibiotics, then they can be switched to oral antibiotics, most commonly metronidazole, for four more weeks [1,3]. Our patient’s early nonspecific presentation that was mistaken for infectious mononucleosis combined with her broad range of symptoms and lack of any diagnostic imaging showing IJV thrombophlebitis greatly delayed appropriate antibiotic treatment. Fortunately, due to the severity of her initial presentation, she was immediately placed on broad-spectrum antibiotics with anaerobic coverage upon hospitalization. Once *F. necrophorum* was identified on culture, she was immediately switched to metronidazole with ceftriaxone to maximize bactericidal activity against *F. necrophorum* and gram-positive cocci while maintaining good CNS penetration.

Anticoagulation is another pillar in the management of LS, but there is little clinical evidence to justify its use. The rare incidence of this syndrome results in limited data for systemic evaluation of the risks and benefits of anticoagulation therapy [5]. Anticoagulation is generally recommended as it increases the rate of breakdown of these pathogenic thrombi and improves penetration of antibiotics into the septic emboli as well [15]. In 2002, a meta-analysis studying the role of anticoagulation on sinus thrombosis found that there was no significant reduction in mortality [18]. A subsequent 2004 prospective study showed that heparin was used for patients with sinus thrombosis 80% of the time, with 79% of patients recovering, 5% of patients experiencing major morbidity, and 8% of patients succumbing to mortality [18]. This has set the standard of care for sinus venous thrombosis, and most neurologists tend to treat with anticoagulation for these cases based on the findings of these studies [18]. For our patient, anticoagulation was started based on this reasoning: to prevent embolization or extension of thrombus as recommended by our hematology and neurosurgery departments.

Surgical intervention is indicated for LS complicated by an abscess formation. It is done to achieve source control, increase penetration of antibiotics, and prevent further production of septic emboli [3]. It has been reported in the literature that patients begin to improve and begin to overcome the infection only after surgical drainage of the pus has occurred [2]. Our patient required multiple sinus surgeries with maxillary antrostomy, ethmoidectomy, sphenoidectomy, and septoplasty before the disease could be managed and clinical improvement could occur.

We recently published a systematic literature review on LS with a focus on ophthalmological complications. In that review, we identified a total of 27 patients between 2009 and 2019 who met our criteria for LS and who presented with "any symptom involving the orbit, the extraocular muscles, the optic nerve, the oculomotor nerve, the trochlear nerve, the abducens nerve, or the cavernous sinus” [20]. As a comparison, the previously mentioned 2016 review by Johannesen and Bodtger identified 137 LS patients over only five years (2010-2015), and they had a very similar inclusion criterion as we did [3]. Our 2020 review found that impaired extraocular motility, which would present as diplopia and cranial nerve III, IV, and VI palsies, was the most common presenting symptom, with 88.89% of LS cases (24 cases) presenting with that ophthalmological complication: abducens nerve palsies specifically occurred in 50% of LS cases (12 cases) with extraocular motility impairment and 44.44% of all LS cases with ophthalmological complications as defined by that criteria [20]. Our patient in this case had many symptoms that would classify her as having LS with ophthalmological complications including diplopia, bilateral abducens nerve palsies, blurred vision, and proptosis. Blurred vision or decreased visual acuity was specifically one of her earliest presenting symptoms along with diplopia. She also specifically had cavernous sinus thrombosis, which occurred in 70.37% (19 cases) of cases in our systematic review, and decreased visual acuity, which occurred in 29.63% (8 cases) of those cases [20]. The majority of cases in our review had imaging that confirmed IJV thrombosis (81.48%; 22 cases) but did not have culture confirming *F. necrophorum* from a sterile site (only 33.33%; 9 cases) [20]. Our patient did not follow this pattern in the literature we reviewed as she actually had positive cultures with negative imaging. Moreover, our patient was treated with both anticoagulation and surgical intervention, which was also used in 51.85% of those cases (14 cases) [20]. Furthermore, the antibiotics used to treat our patient with LS were among the two most commonly prescribed in our review of the literature. Metronidazole was used in 40.74% (11 cases) of cases and ceftriaxone was used in 25.93% (7 cases) of cases [20]. Based on the literature review, it would appear that our case was somewhat atypical for this already rare presentation of LS. Her presenting symptoms and treatment plan were among the most commonly reported for this subset of patients, but her diagnostic tests, specifically blood culture results, and IJV imaging were quite unusual. Despite this relatively unique presentation, we believe that this case brings further light to the exceptionally rare cases of LS where there are significant ophthalmological complications.

To summarize, our patient initially presented with a sore throat and a fever that quickly progressed to severe sinusitis, proptosis, and meningitis. We presume that the *Fusobacterium* from her oropharynx extended into the adjacent sinuses and then underwent hematogenous spread to cause bacteremia. Additionally, this bacteremia then led to multiple septic emboli that resulted in the cavernous sinus thrombosis, superior ophthalmic vein thrombosis, and meningitis, that further complicated this challenging case of LS. The imaging of the brain, positive blood cultures, and CSF findings supported our diagnosis of LS complicated by cavernous sinus thrombosis, abducens nerve palsy, and meningitis. Recommendations from infectious disease, ENT, and ophthalmology services allowed us to optimally treat our patient and coordinate her care such that she received the appropriate IV antibiotics, anticoagulation, and operative management required to minimize any long-term morbidity.

## Conclusions

LS is a rare and potentially fatal illness that requires a high index of suspicion, early diagnosis, and aggressive management with a multidisciplinary approach. There is limited published literature describing CNS involvement in LS. Timely diagnosis and treatment are crucial in preventing morbidity and mortality from severe systemic manifestations. LS, once called the “forgotten disease” because of the low incidence, may not be as uncommon as previously reported. We report this case to increase awareness among practicing clinicians to consider LS as a diagnosis in patients presenting with oropharyngeal infections with systemic symptoms.
